# Dynamics of monocytic HLA-DR expression differs between bacterial etiologies during the course of bloodstream infection

**DOI:** 10.1371/journal.pone.0192883

**Published:** 2018-02-21

**Authors:** Sara Cajander, Gunlög Rasmussen, Elisabet Tina, Anders Magnuson, Bo Söderquist, Jan Källman, Kristoffer Strålin

**Affiliations:** 1 Department of Infectious Diseases, Faculty of Medicine and Health, Örebro University, Örebro, Sweden; 2 Department of Clinical Research Laboratory, Faculty of Medicine and Health, Örebro University, Örebro, Sweden; 3 Clinical Epidemiology and Biostatistics, School of Medical Sciences, Örebro University, Örebro, Sweden; 4 School of Medical Sciences, Faculty of Medicine and Health, Örebro University, Örebro, Sweden; 5 Department of Infectious Diseases, Karolinska University Hospital, Stockholm, Sweden; 6 Department of Medicine, Huddinge, Karolinska Institutet, Stockholm, Sweden; University of Toronto, CANADA

## Abstract

**Objective:**

In the pathogenesis of sepsis, activation of both pro- and anti-inflammatory responses are key components, but knowledge is lacking on the association between bacterial etiology and development of dysregulated responses with sustained immunosuppression. The aim of this study was to evaluate how the immunosupression marker HLA-DR on monocytes (mHLA-DR) is associated with bacterial etiology and markers of inflammation during the clinical trajectory of bloodstream infection (BSI).

**Methods:**

Ninety-one adults, predominantly non-ICU patients, with BSI caused by *Streptococcus pneumoniae* (n = 27), *Staphylococcus aureus* (n = 22), *Escherichia coli/Klebsiella pneumoniae* (n = 23), and other species (n = 19) were prospectively included, and sampled on admission (day 0) and on days 1–2, 3, 7±1, 14±2, and 28±4.

**Results:**

The dynamics of mHLA-DR, measured by flow cytometry, differed significantly between etiology groups (*p*<0.001). Patients with *S*. *pneumoniae* and *S*. *aureus* BSI demonstrated low initial mHLA-DR, with the *S*. *aureus* group showing delayed recovery over time. Eleven patients (55% *S*. *aureus*) had negative outcome (secondary bacteremia or death) and they demonstrated sustained C-reactive protein elevation, neutrophilia, lymphocytopenia, and loss of mHLA-DR.

**Conclusions:**

Dynamics of mHLA-DR varied according to the bacterial etiology of infection, with delayed recovery in patients with *S*. *aureus* BSI. Patients with negative outcome showed sustained CRP elevation, neutrophilia, lymphocytopenia, and low levels of mHLA-DR, supporting the theory of a dysregulated host response with persistent inflammation and immunosuppression in late stages of deleterious sepsis.

## Introduction

During the past decade, it has been recognized that the role of the immune system in sepsis is perhaps more complex than previously imagined [1]. The previous dogma of sepsis pathobiology based on excessive inflammation is insufficient [2, 3]. This is illustrated by repeated failures of interventional trials aiming to alter the trajectory of the disease by blocking pro-inflammatory pathways [4]. In contrast to previous dogma, more recent research has shown that pro- and antiinflammation appear simultaneously in sepsis [1] and that a dysregulated response with sustained inflammation and immunosuppression is linked to development of critical illness with deleterious secondary infections [5, 6]. In an autopsy study, Torgesen et al. found that more than 70% of patients who died from sepsis had unresolved infectious foci despite antibiotic treatment [7]. Two other post-mortem studies of patients who died following sepsis demonstrated findings consistent with profound immunosuppression, including extensive lymphocyte apoptosis [8] and diminished expression of human leukocyte antigen-DR (HLA-DR) in lung and spleen tissue [9]. According to this and to advances in basic research of sepsis pathobiology, the definition of sepsis was updated in 2016 [10]. The underlying pathogenesis is today described as a dysregulated host response to infection [10]. However, the mechanisms leading to dysregulated immune responses with sustained immunosuppression are still not clearly understood. In particular, differences related to the etiology of infection have not been addressed in this context previously. This is important to consider, as different etiological bacterial pathogens have different virulence properties and are thus prone to cause different types of infection, including chronic infections for some pathogens [11].

The HLA-DR expression on antigen presenting cells acts as an important immunological synapse in antigen-dependent lymphocyte activation, and its loss of expression on monocytes is suggested to be a diagnostic and prognostic marker of sepsis-related immunosuppression [12]. Accordingly, expression of monocytic HLA-DR (mHLA-DR) has been proposed to be used as a guide to initiate immunostimulating therapy in sepsis [13]. However, a retrospective intensive care unit (ICU)-study could not identify a suitable single cut-off value of mHLA-DR for prediction of adverse outcome, due to wide variations between individuals [14]. The authors discussed that the different etiologies in their setting could have contributed to the variations. To the best of our knowledge, no previous clinical studies have measured the mHLA-DR expression over time and in relation to bacterial etiology of the primary infection. Therefore, the primary aim of the present study was to evaluate how the dynamics of mHLA-DR expression differ between bacterial etiologies of bloodstream infection (BSI). The secondary aim was to assess how levels of mHLA-DR and commonly used markers of inflammation (C-reactive protein (CRP), neutrophil count and lymphocyte count) are expressed in relation to each other and to the outcome of BSI.

## Materials and methods

### Study design and setting

This prospective study was conducted between 2011 and 2014 at the Department of Infectious Diseases, Örebro University Hospital, Sweden. Blood cultures were collected on hospital admission (day 0) from patients with suspected bacterial infection. The inclusion criterion was blood culture positivity within 1–3 days of admission. Exclusion criteria were prior participation in the study or documented infection with human immunodeficiency virus, hepatitis B or C. Blood samples for neutrophils, lymphocytes, monocytes, CRP-levels, and mHLA-DR expression were collected on days 1–2, 3, 7±1, 14±2, and 28±4 after admission.

Information regarding comorbidities, clinical data, other microbiological data, and outcome data, was obtained from the patient records. Comorbidity was assessed using the Charlson comorbidity score [15].

Acute disease severity was assessed according to the acute changes from baseline Sepsis-related Organ Failure Assessment (SOFA) score on admission [16]. The baseline level was normalized to zero in patients with no co-existing organ failure prior to the onset of infection. Patients with an acute SOFA score increase of ≥2 were defined as septic, according to the Third International Consensus Definitions for Sepsis and Septic Shock (Sepsis-3) [10].

Neutropenia (neutrophil count <0.5 x 10^9^/L) or immunosuppressive medication (methotrexate, chemotherapeutics, or cortisol dosing equivalent to prednisolone ≥20 mg) was considered to constitute immunosuppression prior to infection.

Patients with secondary BSI and/or death 3–60 days after admission were considered to have negative outcome.

### Blood donor control group

Blood samples from 61 healthy blood donors at Örebro University Hospital were randomly collected and used as controls for mHLA-DR. The sex distribution was 75% male (n = 46) and the median age was 50 years.

### Blood cultures

Venous blood was collected in two sets of blood culture bottles, two aerobic and two anaerobic and was incubated using the BACTEC system (Becton Dickinson, Franklin Lakes, NJ, USA). The bacteria were identified to species level by routine diagnostic laboratory procedures. Identification of bacteria was confirmed by matrix-assisted laser desorption ionization-time of flight mass spectrometry (MALDI-TOF MS) (MicroflexLT, Biotyper 3.1; BrukerDaltonics, Bremen, Germany).

### Flow cytometry analysis of monocytic HLA-DR

Blood was sampled in ethylenediaminetetraacetic acid (EDTA) anticoagulant tubes that were immediately placed on ice and transported to the laboratory for flow cytometry analysis of HLA-DR expression on monocytes (CD14^+^ cells), according to the protocol of Docke et al. [17]. The samples were prepared within 4 hours of collection. Antibody staining was performed using QuantiBRITE^™^ Anti-HLA-DR PE*/Anti-Monocyte PerCP-Cy5.5 (BD Biosciences, San Jose, CA, USA) and QuantiBRITE^™^ PE* (BD Biosciences), in accordance with the instructions of the manufacturer. An FC500 flow cytometer (Beckman Coulter, Fullerton, CA, USA) equipped with an argon laser (488 nm) and HeNe laser (633 nm) and EXPO 32 software was used for flow cytometry analysis. Kaluza v.1.2 software (Beckman Coulter) was used for data analysis. The results are expressed as number of antibodies bound per cell (AB/c).

Some of the mHLA-DR results from this cohort of patients and controls, have previously, in part, been presented in evaluation studies of PCR-based diagnostics of HLA-DR [18, 19]. However, the results were not linked to specific etiologies or outcome data.

### C-reactive protein and leukocyte counts

CRP, neutrophil count, and lymphocyte count were analyzed with accredited routine laboratory methods at Örebro University Hospital.

### Statistics

Levels of inflammatory markers are presented as medians with interquartile range (IQR). Analyses of mHLA-DR were performed after logarithmic transformation since the distribution was evaluated as log-normal. Unadjusted and adjusted linear regression was used to evaluate mHLA-DR on day 1–2 in respect to different patient characteristics and bacterial etiology. Linear mixed models for repeated measurements were used to evaluate the dynamic variation in mHLA-DR at different time points unadjusted and adjusted for sepsis-3 (severity) and pre-existing immunosuppression. A heterogeneous, first-order autoregressive correlation structure was chosen due to best model fit, evaluated using Akaike information criteria (AIC). Time was modeled on a continuous scale to evaluate whether the slope of the geometric mean of mHLA-DR over time showed significant interaction with bacterial etiology, indicating different dynamics. The slopes of the mean mHLA-DR changes over time were estimated for different etiologies and stratified for presence/absence of sepsis. Time was also modeled on categorical scale to estimate the mean ratios in mHLA-DR between subpopulations of different factors at each time point. Bonferroni–Holm corrections were performed for adjustments of multiple comparisons over time. Mann-Whitney U-test was used to compare differences in CRP, neutrophil, lymphocyte and monocyte counts between outcome groups at each time point.

Spearman’s rho was used to assess correlations between mHLA-DR and markers of inflammation (CRP, neutrophil count, lymphocyte and monocyte count). A p-value <0.05 was considered statistically significant. Unpaired T-test, with Bonferroni-Holm correction for multiple comparisons was used for pairwise comparison of mHLA-DR between blood donor controls and study patients at each sampling time point. All statistical analyses were performed with SPSS version 22 (IBM Corp., Armonk, NY, USA).

### Ethics

Ethical approval for the study was obtained from the Regional Ethics Review Board of Uppsala, Sweden (approval number 2009/024). A written informed consent was obtained in all cases and controls.

## Results

### Characteristics of the study population

Altogether 116 adult patients with positive blood culture were enrolled in the study. Six patients were excluded, as presented in the flow chart (S1 Fig). Samples for 19 patients were unavailable on days 1 and 2; the remaining 91 patients were sampled within 1–2 days of admission and were eligible for inclusion in the study. There was no systematic reason for missing samples, and no significant difference in baseline data of patients with missing data compared to patients with full sample series (data not shown). Patient characteristics of the study population and baseline laboratory parameters are given in Table 1. Among the 91 study patients, the median age was 70 years and 56% were males. According to the Sepsis-3 definition, 52% of the patients had sepsis on admission. All patients were hospitalized and 13% were admitted to the ICU. The four most frequently detected pathogens in blood culture were *Streptococcus pneumoniae* (n = 27), *Staphylococcus aureus* (n = 22), *Escherichia coli* (n = 18), and *Klebsiella pneumoniae* (n = 5). Nineteen patients were categorized as having “other etiology.” They had bacteremia with: *Enterococcus* species (n = 4), polymicrobial etiology (n = 4), *S*. *agalactiae* (n = 3), Group C or G streptococcus (n = 3), *Salmonella* species (n = 2), *Pseudomonas aeruginosa* (n = 1), *Enterobacter cloacae* (n = 1), and *Haemophilus influenzae* (n = 1). The distribution of infectious foci is presented in Table 2.

**Table 1:** Demographic and clinical characteristics of patients.

Characteristics		Bloodstream infection etiology			
	Total cohort n = 91	*E*. *coli/K*. *pneumoniae* n = 23	*S*. *aureus* n = 22	*S*. *pneumoniae* n = 27	Other n = 19
Median age, yrs (IQR)	70 (62–79)	75 (60–86)	78 (67–82)	70 (62–74)	69 (62–79)
Sex					
Male, n (%)	51 (56)	9 (39)	20 (91)	9 (33)	13 (68)
Female, n (%)	40 (44)	14 (61)	2 (9)	18 (67)	6 (32)
Median SOFA score change (IQR)	2 (1–3)	1 (0–2)	1.5 (1–3)	2 (1–4)	2 (1–5)
Sepsis, n (%)	47 (52)	6 (26)	11 (50)	20 (74)	10 (53)
Immunosuppression prior to infection, n (%)	7 (8)	4 (17)	1 (5)	1 (4)	1 (5)
Intensive care unit admission, n (%)	12 (13)	2 (9)	2 (9)	5 (7)	3 (16)
Median Charlson co-morbidity score (range)	1 (0–8)	2 (0–4)	1 (0–8)	1 (0–8)	1 (0–4)
Congestive heart failure, n (%)	14 (15)	3 (13)	5 (23)	3 (11)	3 (16)
Chronic lung disease, n (%)	7 (8)	0	1 (5)	5 (19)	1 (5)
Diabetes, n (%)	21 (23)	10 (43)	4 (18)	2 (7)	5 (26)
Cancer, n (%)	5 (5)	0	3 (14)	2 (7)	0
Moderate or severe kidney disease, n (%)	6 (7)	2 (9)	3 (14)	0	1 (5)
Connective tissue disease, n (%)	6 (7)	2 (9)	0	4 (15)	0
Median hospital days (IQR)	6 (3–15)	3 (3–6)	14 (9–43)	5 (4–12)	6 (3–19)
Outcome data					
Combined negative outcome, n (%)	11 (12)	1 (4)	6 (27)	1 (4)	3 (16)
Death 3–60 days, n (%)	10 (11)	1 (4)	5 (23)	1 (4)	3 (16)
Secondary bloodstream infection, n (%)	5 (5)	–	2 (9)	1 (4)	2 (11)

Continuous data presented as median and interquartile range (IQR) or range (min–max). Categorical variables presented as numbers and column percentage. SOFA = Sepsis-related Organ Failure Assessment scale.

**Table 2:** Distribution of infectious foci in different groups of bacterial etiology.

Primary focus of infection		Bloodstream infection etiology			
	Total cohort n = 91	*E*. *coli/ K*. *pneumonia* n = 23	*S*. *aureus* n = 22	*S*. *pneumoniae* n = 27	Other n = 19
Urinary tract, n (%)	21 (23)	18 (78)	0	0	3 (16)
Bronchitis, n (%)	2 (2)	0	0	2 (7)	0
Lungs/lower respiratory tract, n (%)	25 (27)	1 (4)	0	22 (81)	2 (11)
Joint/bone,n (%)	8 (9)	0	7 (32)	1 (4)	0
Heart, n (%)	8 (9)	0	5 (23)	0	3 (16)
Central nervous system, n (%)	2 (2)	0	0	2 (7)	0
Other, n (%)	25 (27)	4 (17)	10 (45)	0	11 (58)

Foci defined as “other” include abdominal, skin/soft tissue, catheter-related/stent graft, and unknown foci.

### Etiology of bloodstream infection, SOFA-score, and immunosuppression affects monocyte HLA-DR on days 1–2

To address the effects of BSI etiology on mHLA-DR expression, a linear regression was performed. The median mHLA-DR level on days 1–2 was 17,500 (IQR11,500–32,200) AB/c in the 91 patients with BSI. As demonstrated in Table 3, the etiology of infection, pre-existing immunosuppression, and SOFA score change on admission were independently associated with mHLA-DR levels on days 1–2. The unadjusted models of linear regression showed that *S*. *aureus* and *S*. *pneumoniae* etiology had significantly lower relative mean mHLA-DR compared to *E*. *coli/K*. *pneumoniae* and that a higher SOFA score change was significantly associated with lower relative mHLA-DR. The findings were similar regarding etiology when, adjustment was made for all variables in the table, as possible confounding from SOFA score, comorbidity, age, sex, and pre-existing immunosuppression could be present. Comorbidity assessed by Charlson score ≥1, was not associated with lower mHLA-DR expression in either unadjusted or adjusted analysis. However, preexisting immunosuppression was found to be associated with lower mHLA-DR levels after adjusted analysis. The effects of age and SOFA score change were evaluated both as categorical variables (SOFA score change <2 or ≥2, age <65 years or ≥65 years) and continuous variables with similar results, as shown in Table 3.

**Table 3:** mHLA-DR expression on day 1–2 in patients with potential confounding baseline factors.

Subpopulation factors N = 91		mHLA–DR (AB/c x 10^3^)	Unadjusted			Adjusted		
	n (%)	Median (IQR)	Mean ratio	(95% CI)	p	Mean ratio	(95% CI)	p
**SOFA score change <2**	44 (48%)	24.5 (16.7–41.2)	Ref			Ref		
**SOFA score change ≥2 (Sepsis-3)**	47 (52%)	12.8 (9.5–22.0)	0.60	(0.46–0.78)	<0.01	0.71	(0.54–0.92)	**0.01**
**Comorbidity, Charlson score = 0**	31 (34%)	16.0 (11.2–35.4)	Ref			Ref		
**Comorbidity, Charlson score >1**	60 (66%)	18.3 (11.8–32.1)	0.94	(0.69–1.26)	0.66	0.95	(0.72–1.25)	0.69
**Male**	51 (56%)	16.7 (9.7–35.1)	Ref			Ref		
**Female**	40 (44%)	18.8 (12.6–26.0)	1.04	(0.78–1.39)	0.78	1.06	(0.80–1.41)	0.68
**Age <65 yrs**	30 (33%)	21.2 (15.0–37.0)	Ref			Ref		
**Age ≥65 yrs**	61 (67%)	15.2 (10.8–31.9)	0.75	(0.56–1.01)	0.06	0.86	(0.65–1.14)	0.28
**No immunosuppression prior to sepsis**	84 (92%)	17.8 (11.5–33.6)	Ref			Ref		
**Immunosuppression prior to sepsis**	7 (8%)	16.6 (11.7–22.4)	0.80	(0.47–1.36)	0.41	0.59	(0.36–0.96)	**0.04**
***E*. *coli/K*. *pneumoniae* etiology**	23 (25%)	30.9 (18.3–51.9)	Ref			Ref		
***S*. *aureus* etiology**	22 (24%)	15.4 (10.3–26.9)	0.56	(0.39–0.81)	<0.01	0.61	(0.41–0.90)	**0.01**
***S*. *pneumoniae* etiology**	27 (30%)	12.3 (9.6–16.8)	0.44	(0.31–0.63)	<0.01	0.49	(0.34–0.71)	**<0.01**
**Other etiology**	19 (21%)	22.9 (13.1–41.0)	0.77	(0.53–1.13)	0.18	0.82	(0.56–1.20)	0.30

Adjusted regression with Sepsis-related Organ Failure Assessment (SOFA) score change per unit and age per years on a continuous scale showed similar results (SOFA score change: mean ratio 0.88 (0.82–0.94), p<0.01; immunosuppression prior to sepsis: mean ratio 0.59 (0.37–0.93), p = 0.02; *S*. *aureus* etiology: mean ratio 0.63 (0.43–0.92), p = 0.02; *S*. *pneumoniae* etiology: mean ratio 0.5 (0.35–0.70), p<0.001). AB/c = antibodies bound per cell; CI = confidence interval; IQR = interquartile range; SD = standard deviation; Ref = reference level.

### Monocyte HLA-DR dynamics differ between bacterial etiologies during the course of infection

**Fig 1:**
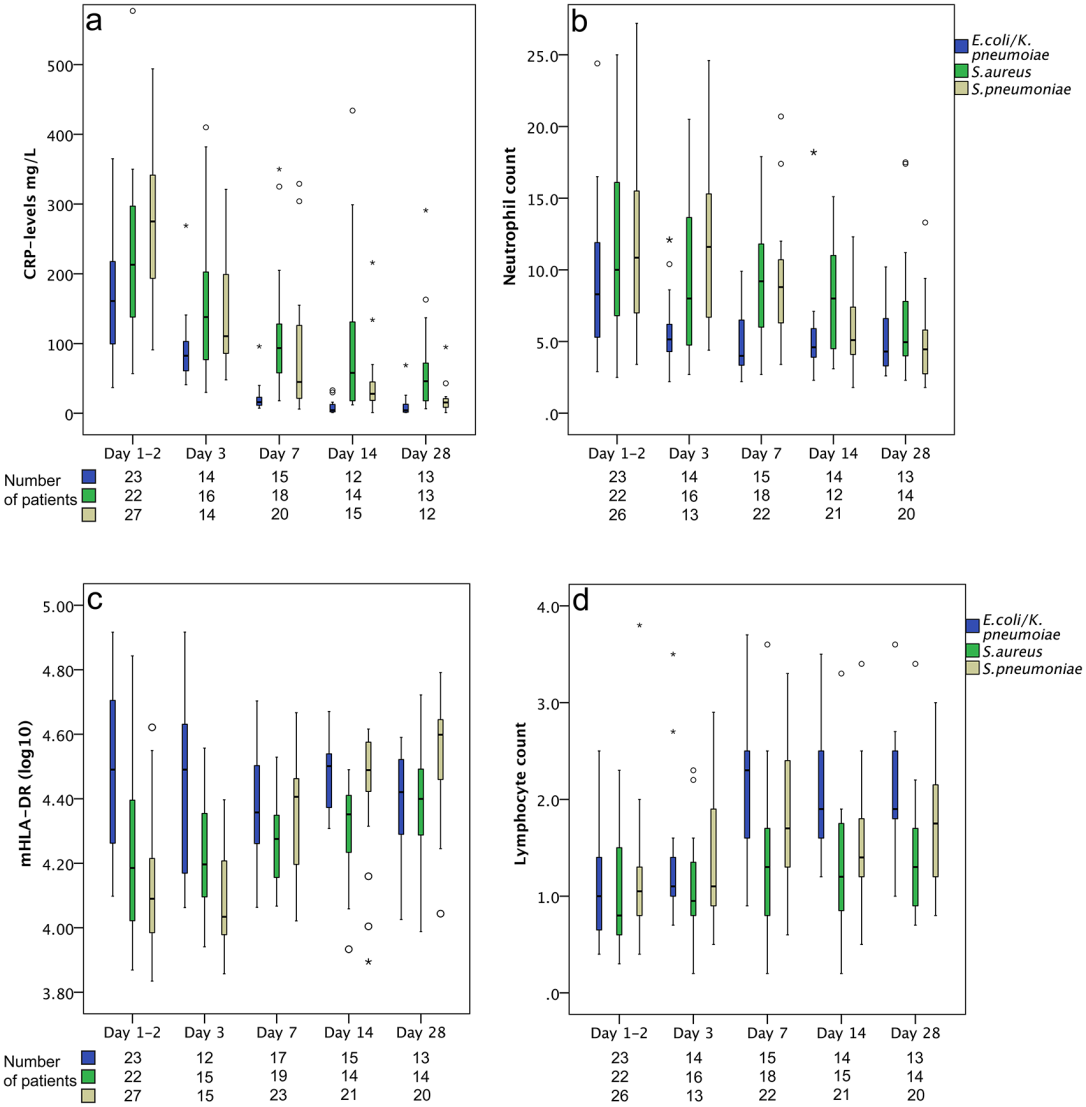
a–d. Dynamic variation of (a) CRP; (b) neutrophil count; (c) mHLA-DR; and (d) lymphocyte count, presented in groups defined by bacterial etiology of bloodstream infection. Box plots give medians (line within the boxes), quartiles (box range), min-max (whiskers) if no outliers were present; otherwise, by circle markers if outliers were more than 1.5 box lengths from the box, and asterisks (*) if outliers were more than three box lengths from the box. The x-axis presents sampling time points, in days after hospital admission.

Monocyte HLA-DR expression was evaluated during the course of infection to study potential differences related to BSI etiology. As shown in Fig 1c, the dynamics of mHLA-DR expression over time differed between the different etiologies of BSI. Patients with *S*. *pneumoniae* BSI demonstrated the lowest initial mean values, with a fast recovery, increasing by 26% per assessment point on average, mean ratio 1.26 (95% CI 1.19–1.33) as estimanted by the linear mixed model. The mHLA-DR levels in patients with *S*. *aureus* BSI demonstrated initial low levels with delayed recovery over time, averagely increasing by 9% between assessment points, mean ratio 1.09 (95% CI 1.01–1.18). By contrast, patients with *E*. *coli*/*K*. *pneumoniae* BSI demonstrated the highest mean mHLA-DR levels without significant mean changes over time. When studying the slopes of mHLA-DR means over time by the mixed model-interaction test, the etiology of BSI was found to differ significantly over time (p<0.001) and remained as a significant factor after multivariate adjustments for Sepsis-3 diagnosis and pre-existing immunosuppression (p<0.001).

As illustrated in Fig 1a the dynamic pattern of CRP and neutrophil counts over time indicated an inverse relation between the dynamics of these markers and mHLA-DR during the course of infection,. However, in contrast to CRP-levels, the neutrophil counts did not demonstrate significant differences between the etiology groups over time (Mixed models interaction test; neutrophil count p = 0.052, CRP p = 0.001). Lymphocyte counts were low in all etiology groups until day 7, as shown in Fig 1d. After day 7, the highest levels were seen in patients with *E*. *coli/K*. *pneumoniae* BSI.

### Delayed recovery of monocyte HLA-DR expression in patients with *Staphylococcus aureus* bloodstream infection

The relative mean differences of mHLA-DR in BSI and blood donor controls are summarized in S1 Table. In comparison to blood donor controls (median mHLA-DR 33,200 AB/c), patients with *S*. *aureus* etiology demonstrated significantly decreased mHLA-DR levels throughout the study period whereas mHLA-DR in patients with *S*. *pneumoniae* were decreased only on the three first sampling time-points. In contrast, patients with *E*. *coli/K*. *pneumoniae* etiology had similar mHLA-DR levels as healthy controls on early assessment points (day 1–2, day 3) and were only slightly decreased on day 7.

### Correlation between monocyte HLA-DR and markers of inflammation

**Fig 2:**
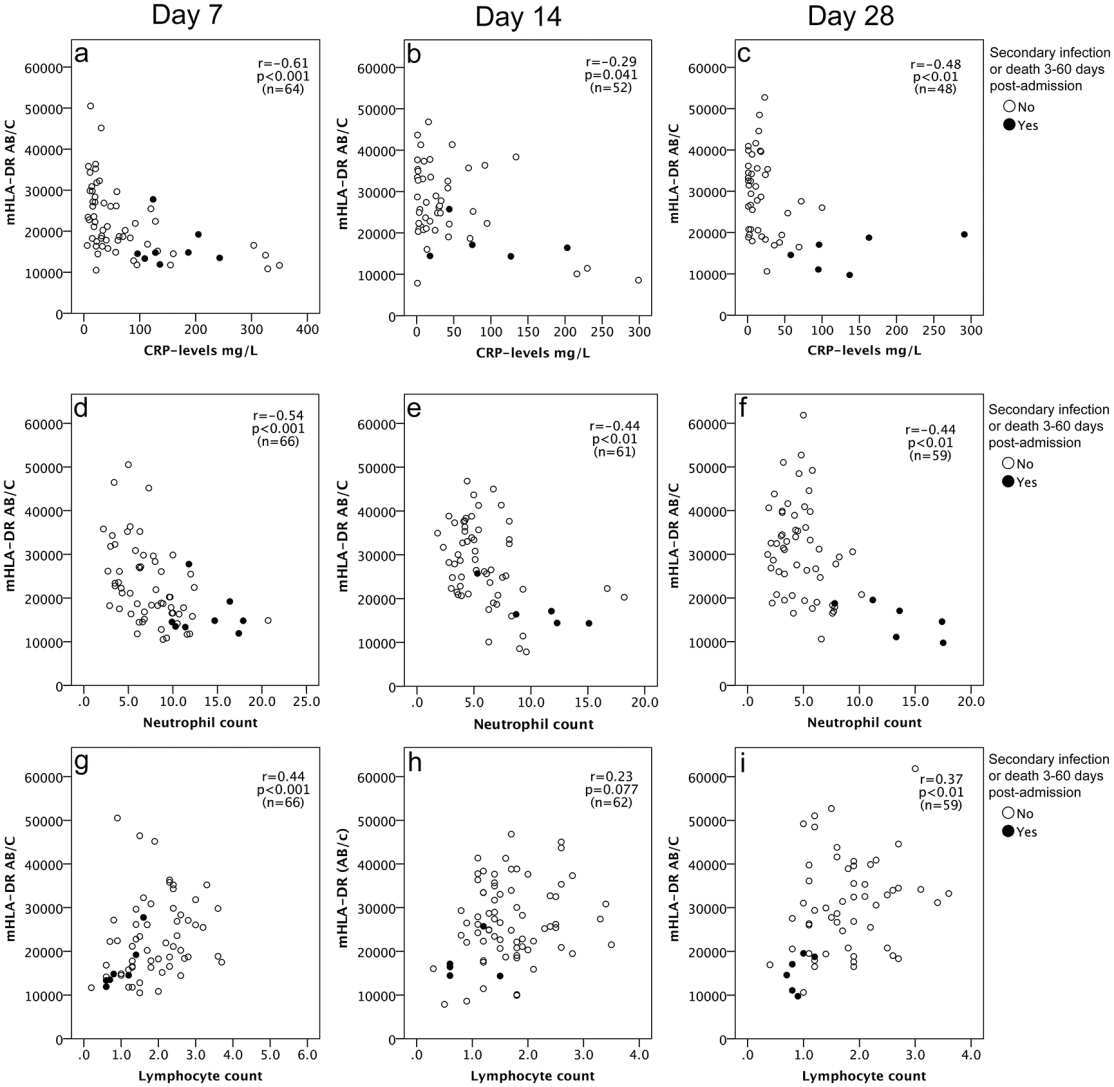
mHLA-DR expression on post-admission days 7, 14, and 28, in relation to CRP (a-c), neutrophil counts (d-f), and lymphocyte counts (g-i), in bloodstream infection with and without negative outcome. Filled circles represent patients with negative outcome. Open circles represent patients without negative outcome.

Monocyte HLA–DR expression demonstrated a weak to moderate inverse correlation to both CRP and neutrophils at all assessment points. Correlations for CRP and neutrophils on the early assessments were on day 1–2; CRP r = -0.29 p<0.01, neutrophil count r = -0.37 p<0.01, on day 3; CRP r = -0.50 p<0.01, Neutrophil count r = -0.56 p<0.01). Fig 2a–2i shows the correlation between mHLA-DR expression and markers of inflammation (CRP, neutrophil count, and lymphocyte count) on days 7, 14, and 28. Lymphocyte counts correlated positively to mHLA-DR, but the correlation was significant only on assessment days 7 and 28 as shown in Fig 2g–2i. By contrast, monocyte levels did not correlate to mHLA-DR expression, except for a weak negative correlation on day 7. Correlations for monocytes were on day 1–2; r = -0.093 p = 0.39, day 3; r = -0.065 p = 0.65, day 7;r = -0.30 p = 0.016, day 14; r = -0.22 p = 0.084, day 28 r = 0.019, p = 0.88).

### Prolonged inflammation, lymphocytpenia and inability to restore mHLA-DR in patients with negative outcome

Altogether eleven of the 91 patients (12%) had a negative outcome 3–60 days after hospital admission, i.e. secondary BSI (n = 5) and/or death (n = 10). Among these eleven patients, *S*. *aureus* was the primary BSI etiology in six patients.

Characteristics of the patients with negative outcome are described in Table 1. Five patients (5.5%) developed secondary BSI and altogether ten patients (11%) died. The SOFA score change on admission was higher in patients with negative outcome; the median score was 5 in the group with a negative outcome and 1 in the group without negative outcome. None of the patients with a negative outcome were immunosuppressed prior to admission.

**Fig 3:**
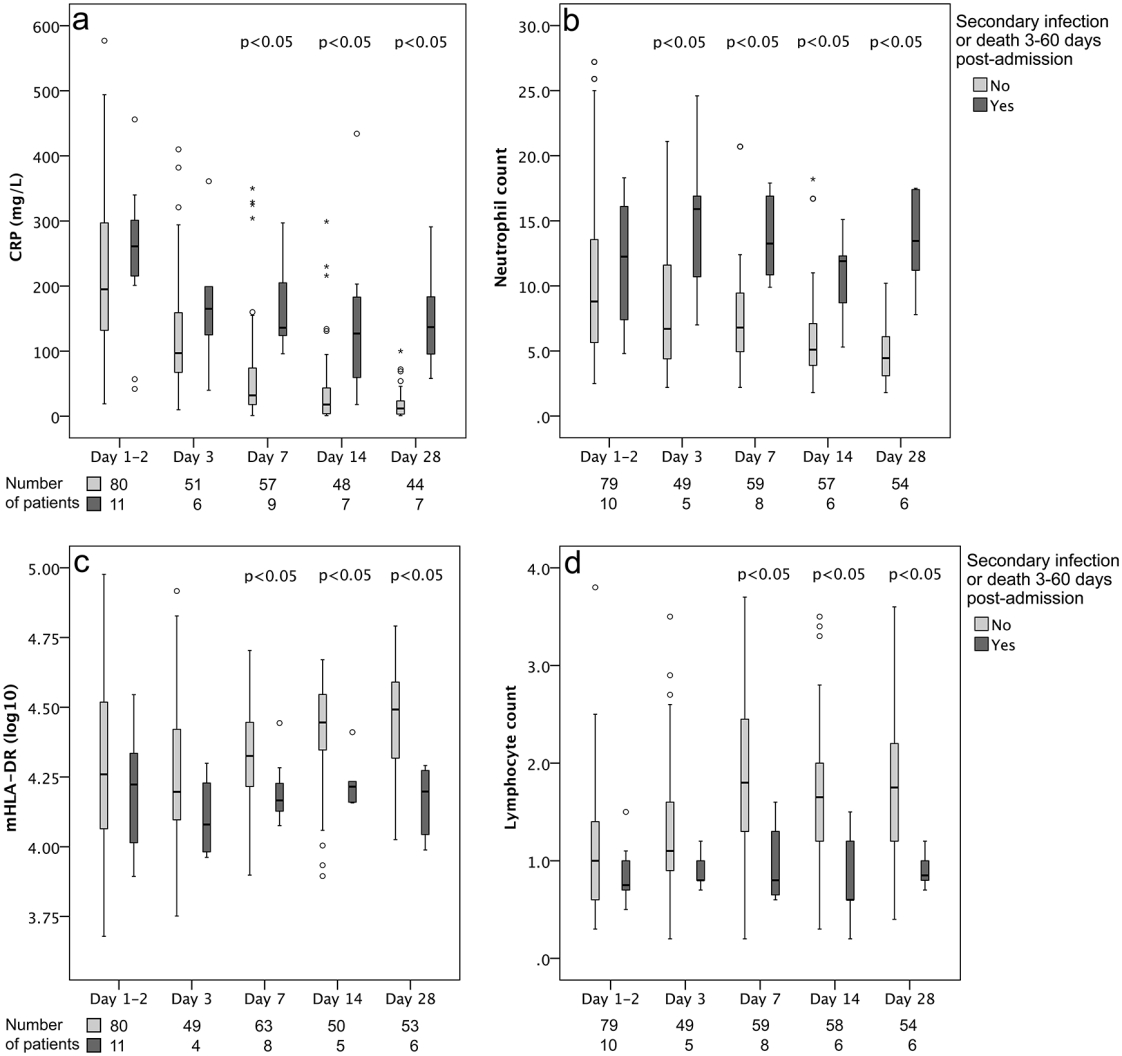
Dynamic variation of (a) CRP; (b) neutrophil count; (c) mHLA-DR; and (d) lymphocyte count, in patients with and without negative outcome (i.e., secondary bloodstream infection [BSI] or death 3–60 days post admission). Box plots give medians (line within the boxes), quartiles (box range), and min–max (whiskers) if no outliers were present, otherwise circle markers if outliers were more than 1.5 box lengths from the box, and asterisks (*) if outliers were more than three box lengths from the box.

Patients with a negative outcome failed to restore mHLA-DR and lymphocyte counts and demonstrated a sustained CRP elevation and neutrophilia in comparison to patients without a negative outcome (Fig 3). Monocyte counts did not differ significantly between outcome groups on any assessment point (S2 Fig).

## Discussion

In this study of patients with community-onset BSI, the dynamics of mHLA-DR differed depending on the bacterial etiology and were inversely associated with dynamics of CRP and neutrophil counts. Patients with negative outcome showed sustained CRP elevation, neutrophilia, and lymphocytopenia, and low levels of mHLA-DR. During the early phase of infection, both *S*. *pneumoniae* and *S*. *aureus* BSI were associated with low mHLA-DR levels, in contrast to *E*. *coli*/*K*. *pneumoniae* BSI. These etiology-related expressions of mHLA-DR remained after multivariate adjustments for baseline factors and the presence/absence of sepsis.

Previous studies evaluating mHLA-DR in sepsis rarely present results in relation to the etiology [13, 20–22], despite knowledge of etiology-related differences in sepsis outcome [23]. In a recently published meta-analysis of translational immunology in intensive care medicine, only one study specified etiology and or/ site of infection [24]. However, in the study by Janols et al. [25], patients with Gram-positive infections expressed lower mHLA-DR compared to patients with Gram-negative infections, similar to our observations in the initial phase of infection. Nevertheless, we also showed important differences in the dynamics of mHLA-DR within Gram-positive etiologies, i.e., between *S*. *pneumoniae* and *S*. *aureus* infections. Patients with *S*. *pneumoniae* BSI demonstrated a rapid recovery after day 3, whereas patients with *S*. *aureus* BSI demonstrated a longer duration of decreased levels (Fig 1). This is probably in part due to the complex interaction between pathogens and host immune defense mechanisms leading to different exposures to damage-associated molecular patterns (DAMPs) and pathogen-associated molecular patterns (PAMPs) during the course of BSI [26]. Indeed, it is well known that *S*. *pneumoniae* is most often rapidly cleared from the bloodstream after initiation of antibiotic therapy, and that recurring infections with persisting bacteremia despite accurate antibiotic treatment are a hallmark of complicated *S*. *aureus* infections [27]. Therefore, in *S*. *aureus* BSI, a prolonged PAMP exposure may cause repeated toll-like receptor-2 stimulation of immune cells, which may drive the immune response towards development of antigen tolerance, with monocyte deactivation (measured by mHLA-DR loss) [28] and subsequent T-cell deactivation [29].

In contrast to the Gram-positive bacteria, *E*. *coli*/*K*. *pneumoniae* BSI did not have diminished mHLA-DR expression or low lymphocyte counts in this setting. Instead, the highest lymphocyte counts were seen in patients with *E*. *coli*/*K*. *pneumoniae* etiologies. The majority of patients with Gram-negative infections in this study had pyelonephritis as the primary focus of infection. Therefore, it is possible that the high mHLA-DR levels shown in *E*. *coli*/ *K*. *pneumoniae* BSI are confounded by the focus of infection. However, urinary tract infection is shown to be an uncommon primary infection in patients who develop secondary infections [30]. This is also supported by the autopsy study by Torgesen [7], in which pyelonephritis was an uncommon site of unresolved septic foci. In contrast to *S*. *aureus* bacteremia, Gram-negative bacteremia is often transient [31] and thus, the PAMP exposure over time is probably lower in Gram-negative BSI than in *S*. *aureus* BSI. In the present study, it should also be noted that a low percentage of *E*. *coli*/*K*. *pneumoniae* patients had sepsis.

Contemporary data suggests that sepsis-induced immunosuppression is linked to persistent inflammation with elevated CRP levels and neutrophil counts along with presence of lymphocytopenia and immature myeloid suppressor cells in peripheral blood [32–34]. This is referred to as the “persistent inflammation-immunosuppression and catabolism syndrome (PICS)” [34]. However, these new insights are debated as the supporting data based on genomics and immunologic phenotyping are gathered from separate studies on trauma and sepsis patients that all have shortcomings [35]. To our knowledge, no previous study has investigated mHLA-DR expression in relation to the simultaneous expression of CRP, neutrophil, and lymphocyte counts during the disease trajectory in patients with BSI. The results from our study support the inflammation-immunosuppression theory by demonstrating that mHLA-DR was inversely related to CRP and neutrophils, and that patients with a negative outcome often show sustained CRP elevation, neutrophilia, lymphocytopenia, and loss of mHLA-DR (Figs 2 and 3).

In line with previous studies [5, 20] most patients with a negative outcome in the present study had suppressed immune function with low mHLA-DR and lymphocyte counts. This indicates that these biomarkers may be promising tools for selection of patients for immunostimulating therapy. So far, there is no consensus about when such therapy should be given or if treatment may be more successful in certain types of infection. In the present study, *S*. *aureus* etiology was associated with a failure to exhibit a trend towards restoration of mHLA-DR values and lymphocyte counts, and was the most common etiology among patients with negative outcome. For identification of patients who would benefit from immunostimulating therapy, stratification based on the etiology of infection and a combination of markers reflecting different mechanisms of immunosuppression would probably be useful, in order to achieve an individualized treatment tailored by the immune status.

This study has some limitations. First, it was based on patients with positive blood cultures. Therefore, the conclusions may not be valid for an unselected population with suspected sepsis. Secondly, the number of patients with a negative outcome was too low to enable adjustment for confounders to evaluate the prognostic value of mHLA-DR and markers of inflammation for prediction of a negative outcome. On the other hand, the strength of this study was that all patients had proven bacterial infections. This enabled studies of the host response to different BSI etiologies. Moreover, this study was not restricted to ICU patients, which allows the results to be applicable for BSI patients of different severity.

In conclusion, the present study showed substantial differences in markers of host responses during BSI, attributable to the etiology of bloodstream infection. Patients with *S*. *aureus* etiology failed to exhibit a trend toward restoration of normal mHLA-DR values, while those with *S*. *pneumoniae* etiology demonstrated a rapid restoration. No signs of monocyte deactivation or lymphocytopenia were seen in patients with *E*. *coli/K*. *pneumoniae* etiologies of BSI. Patients with negative outcome demonstrated a dysregulated immune response with signs of monocyte deactivation, lymphocytopenia and continuous elevation of proinflammatory markers, supporting the theory of persistent inflammation and immunosuppression in deleterious sepsis.

The present study demonstrated important differences in host response related to the bacterial etiology of sepsis. In view of the upcoming refined diagnostics of infectious diseases [36, 37], individualized immunomodulation therapies according to the host response and the bacterial etiology should be a possible future approach to optimize the treatment outcome of a dysregulated host response in sepsis.

## Supporting information

S1 Fig Flow chart. Flow chart presenting numbers of patients included in the study and time points for measurement of mHLA-DR, C-reactive protein (CRP), neutrophil, and lymphocyte counts.
(TIF)

S2 Fig Dynamic variation of monocyte counts, in patients with and without negative outcome (i.e., secondary bloodstream infection [BSI] or death 3–60 days post admission). Differences between outcome groups were non-significant on all assessment points. Box plots give medians (line within the boxes), quartiles (box range), and min–max (whiskers) if no outliers were present, otherwise circle markers if outliers were more than 1.5 box lengths from the box, and asterisks (*) if outliers were more than three box lengths from the box.
(TIF)

S1 Table Monocyte HLA-DR relative mean difference in blood donors and patients with bloodstream infection. (DOCX)
