# Supplementary figures and images for: Dynamics of monocytic HLA-DR expression differs between bacterial etiologies during the course of bloodstream infection
Source: PLoS One. 2018 Feb 21;13(2):e0192883. doi: 10.1371/journal.pone.0192883 (PMC5821339; doi:10.1371/journal.pone.0192883)

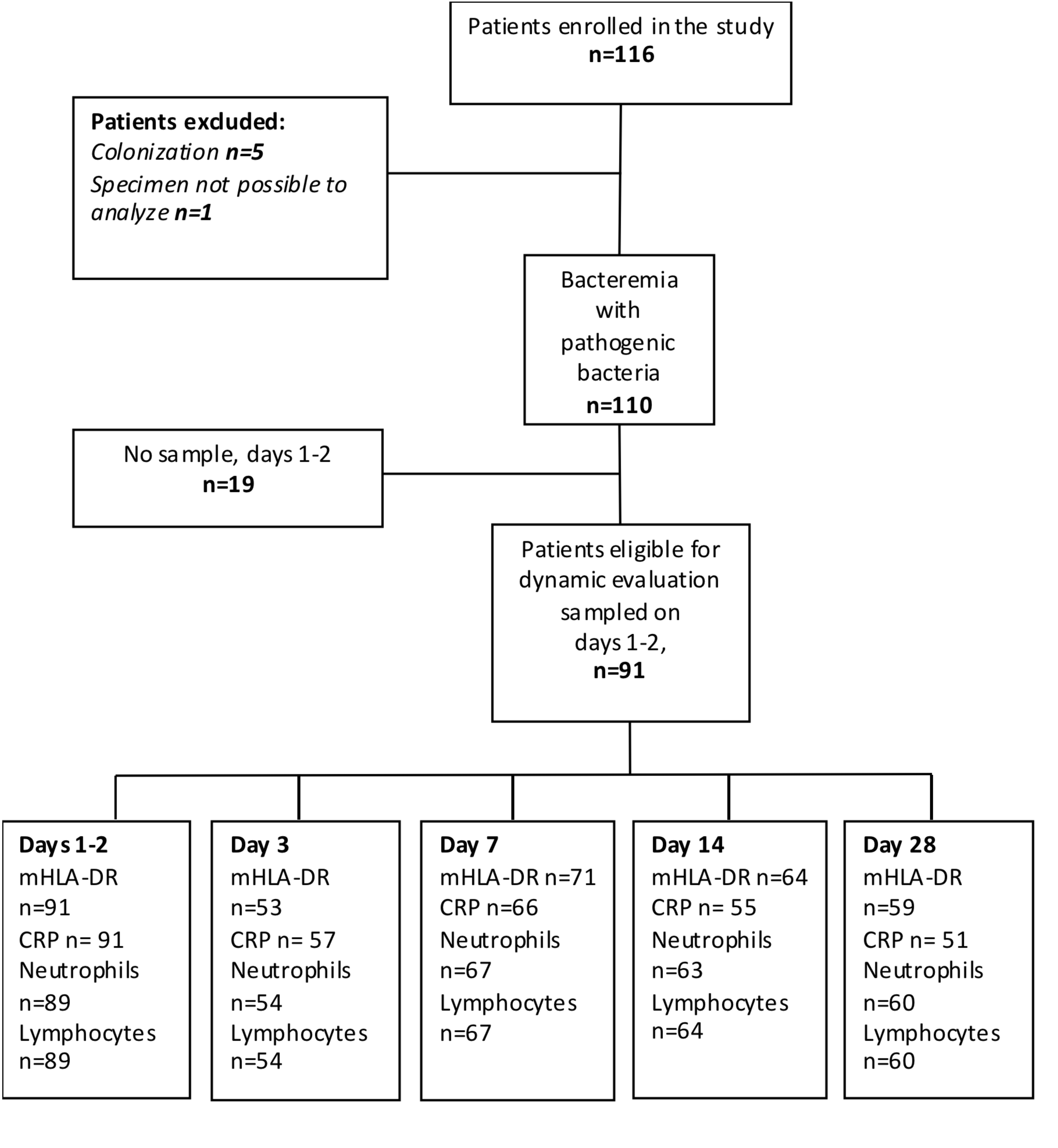

Supplement: S1 Fig — Flow chart presenting numbers of patients included in the study and time points for measurement of mHLA-DR, C-reactive protein (CRP), neutrophil, and lymphocyte counts. (TIF) [file pone.0192883.s001.tif]

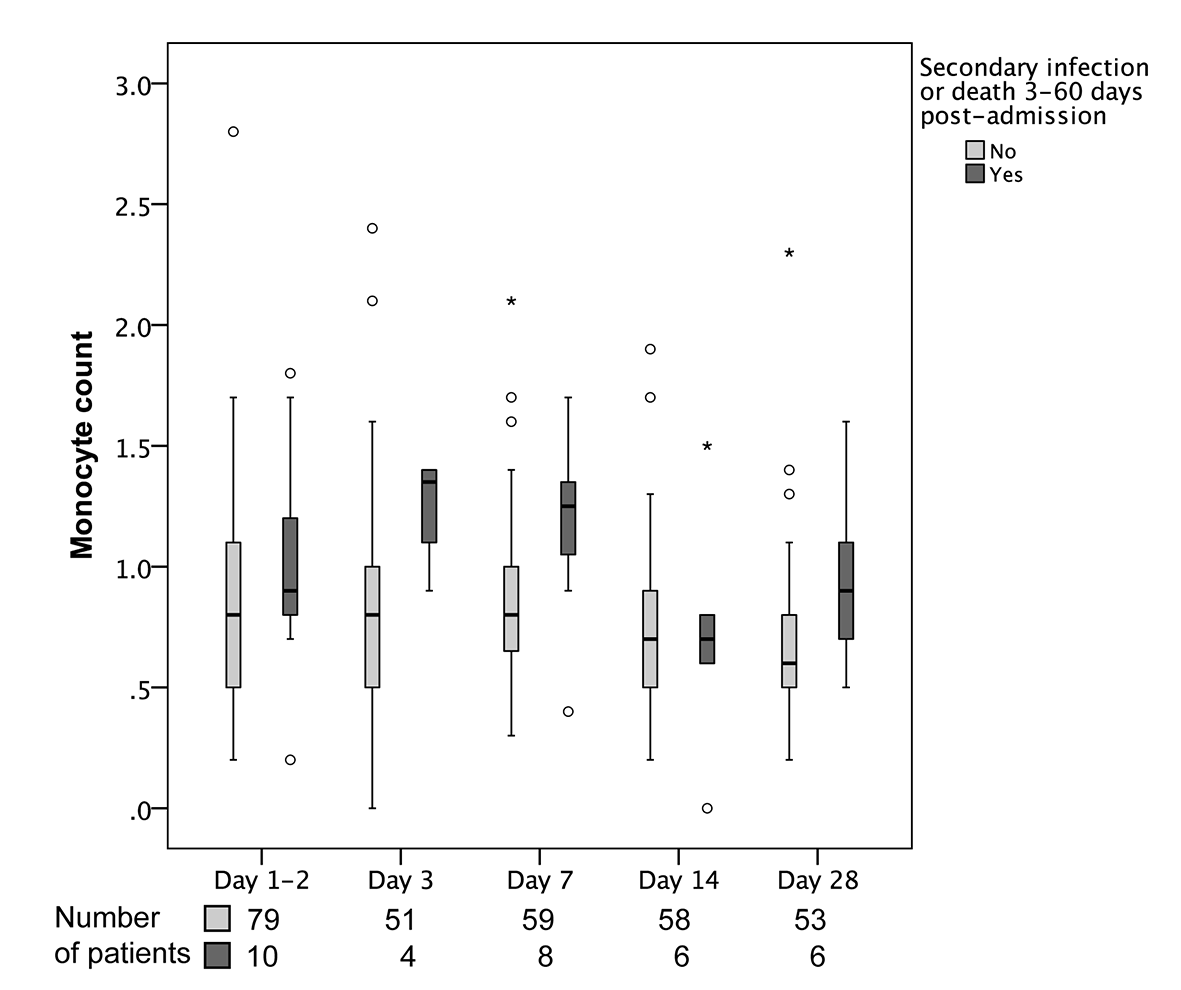

Supplement: S2 Fig — Differences between outcome groups were non-significant on all assessment points. Box plots give medians (line within the boxes), quartiles (box range), and min–max (whiskers) if no outliers were present, otherwise circle markers if outliers were more than 1.5 box lengths from the box, and asterisks (*) if outliers were more than three box lengths from the box. (TIF) [file pone.0192883.s002.tif]
