# Supplementary material for: Dynamics of monocytic HLA-DR expression differs between bacterial etiologies during the course of bloodstream infection
Source: PLoS One. 2018 Feb 21;13(2):e0192883. doi: 10.1371/journal.pone.0192883 (PMC5821339; doi:10.1371/journal.pone.0192883)
Supplement: S1 Table — (DOCX) [file pone.0192883.s003.docx]

**S1 Table. Monocyte HLA-DR relative mean difference in blood donors and patients with bloodstream infection**

|  | ***E. coli/K. pneumoniae*** | | ***S. aureus*** | | ***S. pneumoniae*** | |
| --- | --- | --- | --- | --- | --- | --- |
| Days after admission | mHLA-DR AB/c x 10^3^ median value (IQR) | Mean difference ratio, p-value | mHLA-DR AB/c x 10^3^ median value (IQR) | Mean difference ratio, p-value | mHLA-DR AB/c x 10^3^ median value (IQR) | Mean difference ratio, p-value |
| Day 1-2 | 30.9 (18.3-51.9) | 0.93, p=0.57 | 15.4 (10.5-24.9) | 0.52, p<0.001 | 12.3 (9.6-16.8) | 0.41, p<0.001 |
| Day 3 | 31.0 (14.8-43.6) | 0.85, p=0.16 | 15.7 (12.4-25.6) | 0.51, p<0.001 | 10.8 (9.2-16.7) | 0.39, p<0.001 |
| Day 7 | 22.8 (18.2-31.8) | 0.73, p<0.001 | 18.9 (14.1-22.4) | 0.56, p<0.001 | 25.5 (15.2-29.6) | 0.66, p<0.001 |
| Day 14 | 31.7 (22.3-35.3) | 0.91, p=0.26 | 22.5 (17.1-25.7) | 0.62, p<0.001 | 30.8 (26.5-37.6) | 0.84, p=0.10 |
| Day 28 | 26.3 (19.5-33.3) | 0.75, p=0.096 | 25.1 (19.4-31.0) | 0.73, p<0.001 | 39.7 (28.9-44.2) | 1.04, p=0.69 |
